# Exploring coping strategies among adolescents during COVID‐19 and war displacement: A qualitative analysis comparing two crisis settings

**DOI:** 10.1111/jora.70150

**Published:** 2026-02-15

**Authors:** Sophia Chabursky, Sabine Walper

**Affiliations:** ^1^ Life situations and living environments of children German Youth Institute München Germany

**Keywords:** adolescents, coping strategies, COVID‐19 pandemic, qualitative research, Ukrainian refugees

## Abstract

Adolescence is a critical period for developing coping capacities, yet global crises like the COVID‐19 pandemic and war displacement impose unprecedented stressors that can overwhelm existing resources. This study qualitatively explored and compared how adolescents in Germany (*N* = 20 experiencing pandemic lockdown, aged 11–16; *N* = 25 Ukrainian refugees experiencing displacement, aged 12–18) coped with these distinct adversities. Drawing on an integrated theoretical framework (combining the transactional model of stress and coping with a risk and resilience framework), we analyzed semi‐structured interviews using reflexive thematic analysis to explore the connections among contextual stressors, their impact on resources, and reported coping strategies. Findings revealed that while both crises elicited common coping functions—including adapting routines, emotion regulation, maintaining/rebuilding social connections, and positive reframing—the specific form and feasibility of these strategies appeared to be linked to how each crisis uniquely impacted adolescents' personal, social, and material resources. Crisis‐specific strategies were also identified, which seemed to correspond to the distinct resource challenges associated with pandemic confinement (e.g., purposeful engagement with idle time) versus war displacement (e.g., focus on educational continuity amidst profound loss and acculturative demands). These findings underscore that adolescent coping is a dynamic, context‐dependent process contingent on available resources. Understanding these connections between stressors, resources, and coping is crucial for developing interventions that are both broadly applicable and tailored to the specific challenges adolescents face in diverse crisis situations, considering their developmental needs.

## INTRODUCTION

The 21st century has confronted adolescents with a series of profound global crises, impacting them on an unprecedented scale. At the height of the COVID‐19 pandemic, an estimated 1.6 billion students were out of school (UNESCO et al., 2021). Russia's 2022 invasion of Ukraine has (as of late 2024) displaced an estimated 737,000 children internally within Ukraine, while over 1.7 million Ukrainian children were living as refugees abroad (United Nations Human Rights, 2024). These crises impose a multitude of intense stressors – ranging from social isolation and educational disruption to direct trauma exposure – during a formative period already marked by significant developmental change and evolving coping capacities, all of which can negatively affect adolescents' well‐being (Masten & Motti‐Stefanidi, 2020; Steinberg & Morris, 2001). Understanding the diverse ways adolescents navigate such adversities is paramount for developing effective, evidence‐based support.

While it is known that adolescents employ various strategies to cope with stress, the extreme and often multifaceted nature of large‐scale crises can severely test their existing resources and necessitate distinct adaptive responses (Compas et al., 2017; Masten & Narayan, 2012). Current research has extensively documented the psychological impact of specific crises and cataloged various coping behaviors (e.g., Betancourt et al., 2015; Orgilés et al., 2021). However, there remains a need for comparative research that systematically examines how adolescents cope across different types of crises and, crucially, how the unique contextual stressors and available resources within each crisis are associated with these coping processes. Addressing this complexity is essential for building a more nuanced understanding of the adaptive processes adolescents employ, particularly how coping is linked to the specific resources available in different crisis contexts.

This paper aims to contribute to this understanding by qualitatively exploring and comparing the coping strategies adopted by adolescents in Germany during two major, yet distinct, crises: the COVID‐19 pandemic lockdown and war‐related displacement following the war in Ukraine. We seek to illuminate both commonalities and differences in their coping responses and to understand how these relate to the specific contextual demands and resource landscapes of each crisis.

To lay the groundwork for this investigation, we first conduct a review of the relevant literature on stressors and coping in the context of the COVID‐19 pandemic and war displacement. Afterwards, we present our theoretical framework that guides the analysis of this paper. ‘The Current Study’ section then details this paper's specific aims and research questions, leading into our methodology, findings, and discussion of their implications.

## LITERATURE REVIEW

### Contextual stressors of different crises

During the COVID‐19 pandemic, adolescents were hit by a cluster of stressors that simultaneously strained various domains of their daily lives, impacting their personal well‐being, social connections, and access to material supports. The following studies focused on European and North American samples. Prolonged school closures and an abrupt shift to online learning disrupted daily structure and deprived students of dedicated learning spaces and teacher instruction, undermining self‐efficacy and concentration, challenging personal and material resources (Hammerstein et al., 2021). Restrictions on face‐to‐face contact removed peer interaction and extracurricular activities, weakening social resources that normally buffer stress (Ellis et al., 2020). Families reported increased conflict when several members shared limited space and devices for work and study, reducing both emotional support and material resources like quiet study areas (Fegert et al., 2020). Continuous news coverage and uncertainty about infection added an uncontrollable threat, heightening anxiety for many adolescents (Bridgland et al., 2021). This cluster of stressors simultaneously strained personal (e.g., self‐efficacy), social (e.g., peer support), and material (e.g., learning spaces) resources, illustrating the pandemic's disruption across multiple systems crucial for adolescent development and resilience (Masten & Motti‐Stefanidi, 2020).

For Ukrainian refugee adolescents in Europe, a different stressor constellation became evident. Forced flight uprooted them from schools, neighborhoods, and often with fathers left behind under martial law, stripping key social ties and role models (Brücker et al., 2023). War exposure added direct trauma that taxed emotion‐regulation capacity, thereby challenging personal resources (Gonçalves Júnior et al., 2022). As we have seen for other refugee groups, the stressors do not end upon arrival in another host country, as language barriers, temporary housing, and uncertain legal status limit access to material resources such as stable schooling, healthcare, and private space (Li et al., 2016). Refugees also face social stressors such as discrimination and stigmatization (Dow, 2011). Furthermore, they often bear the added burden of concern for the safety of family and friends remaining in conflict zones (Fazel & Stein, 2002), thus eroding personal resources. Specific to the situation of Ukrainian refugees arriving in Germany, many students faced “double schooling,” meaning attending German classes while continuing Ukrainian curricula online. As school attendance is mandatory in Germany, Ukrainian refugee students initially attended so‐called welcome classes consisting solely of Ukrainian refugee students, which focused primarily on language acquisition. At the same time, many adolescents had the desire to progress in their school education as well as stay connected with their home system and secure future opportunities, therefore continuing their Ukrainian schooling. This however significantly increased their academic workload and time pressure (Chabursky et al., 2024; Chabursky & Walper, 2025). This constellation of premigration trauma, displacement challenges, and postmigration stressors aligns with integrative risk and resilience models emphasizing the unique acculturative demands and adaptation pathways of immigrant‐origin youth (Suárez‐Orozco et al., 2018). These demands are central to acculturation theory, which conceptualizes the process of navigating between one's heritage culture and a new host culture as a major source of stress, but also as a critical context for adaptation and identity development (Berry et al., 2006).

Pandemic stressors centred on isolation and loss of routine, while displacement stressors added trauma and systemic uncertainty, thereby eroding personal resources more strongly. Both crises disrupted education and peer contact, but the pandemic primarily limited existing social networks, whereas displacement necessitated adolescents to rebuild networks from scratch while coping with trauma and legal uncertainty. Material resource loss also diverged: pandemic students retained family and legal stability, while refugee students often lost housing, income, and local language competence.

### Coping strategies during distinct crisis situations

During the COVID‐19 pandemic, European adolescents mobilized a diverse array of supports and skills to navigate the lockdown. Establishing study timetables, planning online lessons, and tackling homework were the dominant problem‐focused responses; these relied on cognitive assets such as self‐efficacy and executive skills, but also on material scaffolds – quiet rooms, laptops, and stable broadband (Dąbkowska et al., 2021; Orgilés et al., 2021; Türk et al., 2021; Vallejo‐Slocker et al., 2022). When distancing rules severed face‐to‐face contact, adolescents switched to emotion‐focused strategies that preserved their social resource base: video‐calls, group chats, and joint gaming kept peer intimacy alive and supplied real‐time emotional support (Branquinho et al., 2020; Demkowicz et al., 2022). Physical exercise at home, creative hobbies, healthy eating, meditation, and yoga further regulated mood while bolstering a sense of competence – tactics powered by material resources (sports equipment, musical instruments) and personal regulatory skills (Ogueji et al., 2022). Finally, adolescents engaged in meaning‐focused coping such as positive reinterpretation and hope‐cultivation, drawing chiefly on personal meaning‐making capacities. This was reinforced by encouraging social narratives, which helped sustain well‐being under prolonged uncertainty (Ogueji et al., 2022; Orgilés et al., 2021; Vallejo‐Slocker et al., 2022).

For Ukrainian refugees in Germany pursuing education, learning German, and attending local “welcome classes” while continuing Ukrainian studies online was identified as the dominant problem‐focused response (Chabursky & Walper, 2025). As research on the coping of Ukrainian refugee adolescents is sparse, we also examined the coping of other war‐displaced adolescent refugees. During flight and resettlement, schooling and study‐planning became the linchpin of problem‐focused coping: war‐affected Syrian and Iraqi youths in Turkey and Germany organized homework schedules, honed language skills, and sought tutorials to protect their academic futures (Akgül et al., 2021; Nilles et al., 2022). These tactics leaned on personal assets (self‐efficacy, executive control) as well as material scaffolds – access to schools and classrooms, laptops, steady Wi‐Fi. Emotion‐focused strategies, which relied on their social and cultural resource base, were also frequently reported: Somali Bantu, Bhutanese, and Middle‐Eastern youths in the United States turned to family, community mentors, and culturally familiar counseling to soothe anxiety (Betancourt et al., 2015). Furthermore, maintaining optimism and cultural identity were also found to be helpful responses in mixed‐origin refugee and asylum‐seeking adolescents in the UK and Ireland, albeit necessitating a high activation of personal resources (Maegusuku‐Hewett et al., 2007; Raghallaigh & Gilligan, 2010).

Across studies, adolescents in both crises combined problem‐focused efforts aimed at restoring routine or education with emotion‐ and meaning‐focused efforts that rebuilt social belonging and personal hope. The balance of strategies differed, however: pandemic youths relied on existing digital networks, whereas refugee youths first needed to recreate material and social resources before similar digitally mediated or resource‐intensive coping could occur.

## THEORETICAL BACKGROUND

### Stress and coping during adolescence

The literature reviewed above highlights the complex array of challenges and responses adolescents face in these crises. To systematically analyze these dynamics and understand the mechanisms linking stressors to specific coping responses, we draw on several theoretical frameworks. We begin with the Transactional Model (Lazarus & Folkman, 1987). In this framework, a stressor is defined as any demand that calls for adaptation. Coping strategies are the specific efforts adolescents use when they evaluate these demands through two key appraisals: a primary appraisal of how threatening or challenging the situation is, and a secondary appraisal of whether their available resources match the demands of the threat. The resulting strategies can be broadly classified as problem‐focused (aimed at changing the situation), emotion‐focused (aimed at regulating feelings), or meaning‐focused (aimed at reinterpreting the situation) (Park & Folkman, 1997). Because appraisals are subjective and available resources may differ, individuals facing the same stressor may adopt very different coping responses.

Adolescence marks a distinct developmental stage in which three overarching tasks dominate: gaining behavioral and emotional autonomy within the family system, forging identity commitments (values, vocational plans, gender/ethnic identity), and building mutually supportive peer relationships (Branje et al., 2021; Steinberg & Morris, 2001; Zimmer‐Gembeck & Collins, 2003). These tasks unfold alongside rapid growth in resources. Executive functions and abstract reasoning accelerate, boosting planning and problem‐solving capacity (Casey, 2015). Expanding and more reciprocal peer networks provide additional social support and emotion‐regulation opportunities, while motivational systems become more sensitive to future‐oriented goals (Blakemore & Mills, 2014).

At the same time, adolescents encounter stressors that are broader and more complex than those of childhood: heavier academic demands and high‐stakes examinations, shifting peer hierarchies, status pressure and potential victimization, evolving family expectations, and the existential work of forming a coherent identity (Hair et al., 2008; La Greca & Harrison, 2005; Steinberg & Morris, 2001; Suldo et al., 2009). To handle these everyday stressors, adolescents most often use a mixed coping repertoire. The most common responses are active problem‐solving, seeking emotional support from close friends or family, cognitive distraction through media or hobbies, and re‐appraisal or acceptance (Compas et al., 2017; Zimmer‐Gembeck & Skinner, 2016). Thus, the very processes that make adolescence a period of heightened vulnerability also furnish a growing toolkit of personal and social resources for coping.

### Risk and the process of adaptation in high‐magnitude adversity

Adolescents are not only challenged by day‐to‐day hassles, but some also confront high‐impact adversities such as bereavement, displacement, or large‐scale disasters. Research in these contexts often focuses on resilience, defined as the phenomenon of positive adaptation despite significant adversity (Masten & Narayan, 2012). Masten's multisystem risk and resilience framework, a cornerstone of this field, seeks to understand the individual differences in developmental outcomes following risk exposure, with a focus on why some youth fare well while others do not (Masten et al., 2021; Masten et al., 2023). The framework posits that these outcomes depend on the balance between risk factors that elevate the probability of maladjustment (e.g. chronic economic stress, family separation, forced migration) and protective factors that keep development on track (Masten & Motti‐Stefanidi, 2020; Suárez‐Orozco et al., 2018).

Crucially, the framework distinguishes between resilience as a developmental outcome (i.e., positive adaptation) and the process of adaptation through which it is achieved. To understand why outcomes vary, the framework directs attention to the underlying adaptive processes that produce them. This adaptive process is fueled by the mobilization of protective factors, which take the form of resources that operate at several levels: personal (skills, self‐efficacy, psychological resilience) (Benight & Bandura, 2004), social (support from family, friends and community networks) (Taylor, 2011), and material (financial security, stable housing, access to education) (Hobfoll et al., 2007). Therefore, individual differences in the availability and mobilization of these resources are theorized to give rise to different adaptive pathways – including the use of specific coping strategies – which in turn lead to varying developmental outcomes (Masten et al., 2021; Masten et al., 2023).

However, risk cascades (e.g., displacement) can strip social, material, and personal assets, narrowing the coping options left (Masten & Cicchetti, 2016; Masten & Narayan, 2012). Resource erosion therefore helps explain shifts in coping style under high‐magnitude or uncontrollable events. Meta‐analytic evidence shows that adolescents pivot from problem‐focused tactics toward emotion‐ and meaning‐focused strategies when control is low and resources have been stripped away (Compas et al., 2017; Masten & Narayan, 2012). Coping‐flexibility studies likewise document strategic switching as threats intensify or new supports appear (Cheng et al., 2014).

Taken together, the two theories provide a comprehensive framework for exploring the adaptive process. The multisystem risk and resilience model identifies the critical role of resources as protective factors, while the transactional model illuminates the cognitive and behavioral processes (i.e., appraisal and coping) that adolescents use to navigate their circumstances.

**FIGURE 1:**
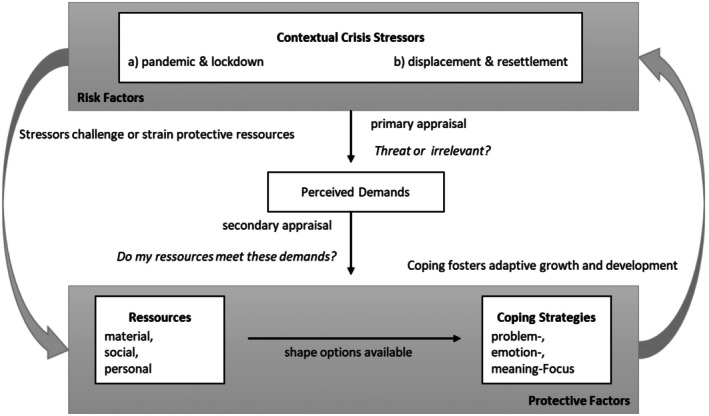
Conceptual model integrating the transactional model of stress and coping with a risk and resilience framework.

This integration is visualized in Figure 1. Contextual stressors (e.g., pandemic lockdown, war displacement; upper box) are understood to erode the personal, social, and material resources (left gray arrow). Adolescents appraise the altered situation and, given the resources still at hand, adopt problem‐, emotion‐, or meaning‐focused coping (lower box). Effective coping can in turn blunt the stressor's impact and foster adaptive growth (right gray arrow). This feedback arrow represents how coping strategies can alter the adolescent's experience of the stressor. While an individual cannot change the macro‐stressor (e.g., the pandemic), their coping actions can directly modify the proximal, personal stressors or buffer the negative emotional impact of the stressor.

For the current study, we use this integrated model as an analytical lens. While we have visualized the process with directional arrows to stay true to the foundational theories, our methodology explores the associations between these components rather than testing directionality. The model, therefore, serves as an interpretive heuristic that guides our analytical process, which involves: (a) interpreting the key stressors evident in the adolescents' narratives; (b) analyzing the associated impact on their resource landscapes; and (c) illuminating the relationship between these available resources and the coping strategies they described.

## THE CURRENT STUDY

Most studies of adolescents in crisis document stressors and catalogue coping strategies, yet few have used a theoretical framework to systematically explore the connections between them. Pandemic studies, for instance, often list stressors like school closures and social isolation without linking them to specific coping responses, and refugee studies often describe war‐related trauma or resettlement challenges but do not always explicitly explore the connections between these specific stressors and the coping strategies employed. As a result, while findings from the COVID‐19 pandemic and Ukrainian displacement show similar strategies – like maintaining routines or seeking peer contact – it remains unclear whether this overlap reflects broadly adaptive adolescent habits or simply the limited set of responses feasible when resources are lost. A theory‐guided comparative study is therefore needed to illuminate these connections.

The present study addresses this gap using descriptive, qualitative data. Given its cross‐sectional design, this study does not measure long‐term resilient outcomes or test causal pathways. Instead, its aim is to provide a rich, contextualized understanding of how adolescents cope with these distinct negative experiences by focusing on the relationship between their available resources (protective factors) and their reported coping strategies.

To achieve this, our inquiry has a dual focus. First, at the individual level, we aim to understand the process of coping by exploring how each adolescent's unique resource landscape is associated with the specific strategies they report. Second, at the group level, we conduct a comparative analysis to understand the impact of context. To do this effectively, we deliberately compare two qualitatively different crises: the pandemic, which posed a chronic, low‐control threat that disrupted daily routines but left core stability intact, and war displacement, which combined acute trauma with the profound loss of social, material, and personal resources. This “most‐different” comparative design allows us to explore which coping dynamics appear to be context‐specific and which may be more broadly functional, thereby offering clearer guidance for practitioners and policymakers.

This dual‐focused, comparative inquiry is guided by the following research questions:

1. What coping strategies do adolescents report in response to the COVID‐19 lockdown and, separately, to war‐related displacement?
2. In each crisis, how are the specific stressors associated with the adolescent's personal, social, and material resources, and how do these resources relate to the coping strategies adolescents report employing?
3. Which coping strategies – and the resource dynamics associated with them – are shared across the two crises, and which appear to be crisis‐specific?

## METHOD

### Study design

The research presented in this paper is a secondary analysis employing anonymized interview data from two qualitative studies conducted at the German Youth Institute. The first original study, conducted in Germany during the spring of 2020 and fall of 2021, focused on the experiences of children, adolescents, and their parents amid COVID‐19 lockdowns. For the present analysis, only adolescent interviews were used. These interviews were conducted in German by two experienced researchers from the German Youth Institute. Interviews in 2020 were conducted via telephone, while those in 2021 were in person, adhering to hygiene protocols. The semi‐structured interview guide spanned topics like pandemic‐related changes in daily life, schooling, social interactions, and psychological well‐being, including questions about mood improvement strategies and dealing with pandemic challenges (e.g., “What has changed in your life since the pandemic?”; “Are there any activities that help you deal with the situation?”). COVID‐19 study interviews lasted approximately 40 minutes, and participants received a 20 Euro voucher.

The second original study, undertaken in fall 2022, explored the experiences of Ukrainian refugee adolescents resettling to Germany. These interviews were conducted in Ukrainian by the first author of the present paper, a native speaker of Ukrainian, German, and English with prior experience in conducting qualitative interviews with children and adolescents. The semi‐structured interview guide covered their escape from Ukraine, adaptation in Germany, schooling (German and Ukrainian), social contacts, leisure, mental health, and future plans, including questions about mood, worries, and coping strategies (e.g., “How did you feel when you first arrived in Germany?”; “How do you deal with your worries?”). Ukrainian refugee study interviews lasted approximately 1 h, and participants received a 30 Euro voucher. The Ukrainian interviews were transcribed verbatim and then translated into German. The first author, given her native fluency in both languages and direct involvement in data collection, reviewed all translations for accuracy and conceptual equivalence, ensuring the fidelity of the data for analysis.

At the time these two original qualitative studies were conducted, the German Youth Institute had not yet constituted a formal Ethics Committee; consequently, no formal approval ID can be provided for the original data collection. Nevertheless, both original studies adhered to the ethical research guidelines of the institute, consistent with standard ethical practice. Accordingly, written informed consent was obtained from parents or legal guardians for all minor participants, and age‐appropriate assent was obtained directly from the adolescent participants themselves. Consent materials explicitly permitted anonymized transcripts to be archived and reused in future research studies, such as the current secondary analysis. Data were encrypted, stored on secure servers, and irreversibly de‐identified. Consent materials emphasized voluntariness, the right to skip any question, and unconditional withdrawal rights. Interviewers prioritized participant well‐being, particularly for the Ukrainian refugee sample, occasionally refraining from probing into sensitive subjects. Furthermore, participants were also provided with information about available psychological support services, should they experience distress following the interviews.

### Data analysis

For this secondary analysis, our focus was primarily on the perspectives of adolescents. From the first original study (COVID‐19), parental interviews were excluded, and participants younger than secondary school age were also excluded to align with the age range of the second sample. This resulted in a final sample for the present study comprising 20 individuals aged 11 to 16 (9 male, 11 female) from the COVID‐19 study and 25 Ukrainian refugees aged 12 to 18 (11 male, 14 female). Detailed sociodemographic characteristics are presented in Table 1.

**TABLE 1:** Sociodemographic characteristics of participants.

Characteristics	COVID‐19 sample (*N* = 20)	Ukrainian refugee sample (*N* = 25)
Age (years)		
Mean (SD)	12.35 (1.81)	14.64 (1.87)
Range	11–16	12–18
Gender		
Female, *n* (%)	11 (55%)	14 (56%)
Male, *n* (%)	9 (45%)	11 (44%)
Has siblings		
Yes, *n* (%)	17 (85%)	15 (60%)
Family receives state support^a^		
Yes, *n* (%)	5 (25%)	12 (48%)

^a^ Note: State support refers to receiving government welfare benefits.

We employed Braun and Clarke's (2022) reflexive thematic analysis for this secondary analysis. The process began with data from the original studies, both of which had undergone an initial phase of coding. For the COVID‐19 study, three researchers collaboratively developed a codebook and coded the data, establishing inter‐coder agreement through independent coding of a subset of four interviews followed by discrepancy resolution discussions. A similar process involving three researchers (including the first author) and a subset of three interviews was used for the Ukrainian refugee study. In both instances, coders received training on the respective codebooks, and regular meetings ensured coding consistency.

Building upon this foundational understanding, the first author of the present paper conducted the in‐depth reflexive thematic analysis for the specific comparative aims of this study. This involved an iterative process, starting with deep re‐familiarization with the selected adolescent transcripts from both datasets. She then systematically re‐coded the data relevant to the current study's research questions, moving beyond the original broader codes to focus specifically on contextual stressors, resource impacts, and coping strategies. This re‐coding was explicitly guided by our integrated theoretical framework (Figure 1). This framework served as an interpretive lens through which we analyzed participants' narrative experiences to identify and label key contextual stressors, their impact on personal, social, and material resources, and the function of their coping responses (problem‐, emotion‐, or meaning‐focused coping).

Following this, identified codes were grouped into potential themes for each dataset separately. The first author engaged in extensive memoing during this phase, documenting analytical insights, interpretations of patterns, the evolution of thematic ideas, and reflections on her positionality (as further detailed in the “Positionality and Reflexivity” section). She also created summaries for each interview to capture holistic impressions. Themes were then refined through rigorous review against the entire dataset and coded extracts, ensuring coherence and distinctiveness. The formation and clustering of these themes were also theoretically informed, considering, for example, the types of resources mobilized by the coping strategies and their primary function (problem‐, emotion‐, or meaning‐focused). During this iterative process of within‐crisis theme development, the first author mindfully noted any emerging awareness of potential cross‐crisis commonalities in her memos to ensure distinct theme construction prior to formal comparison. Only after themes were robustly developed for each crisis context independently did the formal comparison between the two datasets occur. This cross‐crisis comparison aimed to identify shared and crisis‐specific coping strategies and their underlying resource pathways.

To ensure analytic rigor and trustworthiness throughout the reflexive thematic analysis conducted by the first author, regular and extensive discussions were held with the second author. The second author, an experienced researcher, reviewed emerging themes, challenged interpretations, and provided critical feedback. When differing views on data interpretation arose, decisions were made through a dialogic process of re‐examining relevant data extracts, debating nuances of meaning in relation to participants' broader narratives and the theoretical framework, and refining theme definitions until consensus was reached. This collaborative dialogue was crucial for enhancing the depth and credibility of the analysis. Direct quotations from participants are incorporated in the results to bolster the validity of our interpretations, and all participant names are pseudonyms.

### Positionality and reflexivity

Our study is grounded in a constructivist epistemology, which acknowledges that knowledge is co‐constructed between researchers and participants. We chose reflexive thematic analysis as outlined by Braun and Clarke (2022) because it allows for an in‐depth exploration of participants' experiences while recognizing the active role of the researcher in interpreting data.

The research team for this secondary analysis comprised two female researchers whose diverse backgrounds informed our approach. The first author brings a background in health psychology, expertise in psychoregulation, and shares experiences of migration and Ukrainian heritage. This positionality afforded a deep contextual understanding, particularly for the Ukrainian refugee data which she also originally collected, but simultaneously necessitated heightened reflexivity to ensure balanced interpretation across both datasets. The second author, an experienced professor in youth and family studies, contributed significantly to the theoretical framing and provided critical oversight, helping to ground interpretations in established developmental theory.

Acknowledging these potential influences, we engaged in specific reflexive practices throughout this secondary analysis. The first author utilized memoing extensively during her re‐coding and theme development process, documenting analytical decisions, personal reflections, and potential biases in relation to the data. Furthermore, regular discussions between both authors served as a crucial reflexive tool. These discussions were not only for resolving interpretive differences (as detailed in Data Analysis) but also for critically examining how our individual perspectives and the study's evolving theoretical lens were shaping the analysis, allowing us to challenge assumptions and strive for interpretations that were deeply grounded in the participants' accounts.

## RESULTS

**FIGURE 2:**
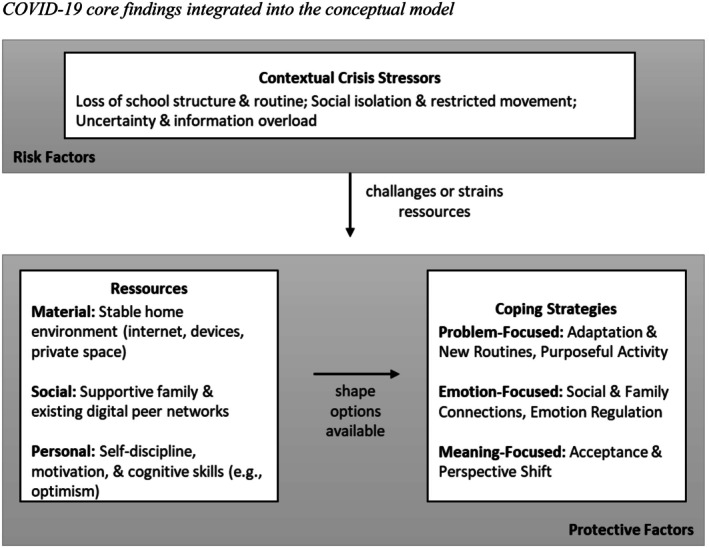
COVID‐19 core findings integrated into the conceptual model.

**FIGURE 3:**
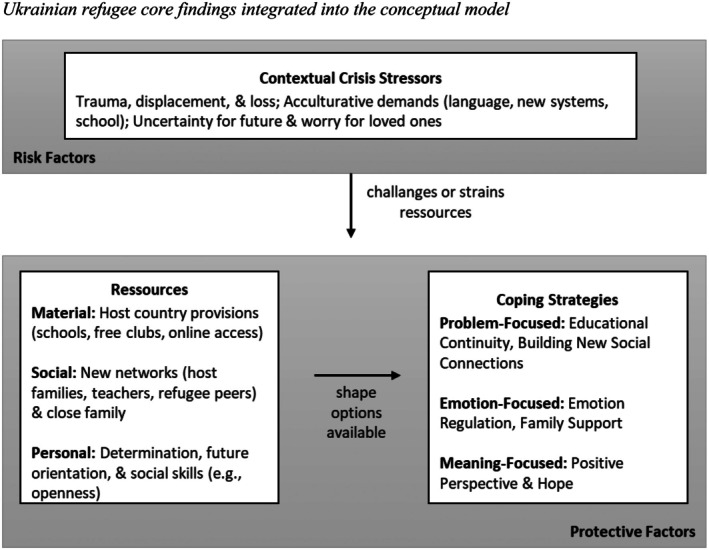
Ukrainian refugee core findings integrated into the conceptual model.

This section presents a detailed analysis of the coping strategies employed by adolescents during the COVID‐19 pandemic and by Ukrainian refugee adolescents. Our analysis followed the logic of our integrated conceptual model, exploring the associations among the interpreted contextual stressors, the resources adolescents mobilized, and their subsequent coping strategies. To provide a clear visual overview of our findings, we first present a summary figure for each group (Figures 2 and 3), shown before the corresponding group’s results, that populates our conceptual model with the dominant coping dynamics identified in the analysis. The arrows within the figures represent conceptual links rather than causal directionality. Following these figures, we delve into the detailed thematic analysis for each group. For a comprehensive tabular overview of all themes, including specific stressors, associated resources, and each theme's prevalence, see Table S1.

### COVID‐19 findings and themes

Figure 2 visually summarizes the dominant associations identified for the COVID‐19 group, mapping the key stressors and mobilized resources to the overarching coping focus. The specific themes characterizing this dynamic are detailed below.

#### Adaptation and new routines

The sudden shift to remote learning under lockdown meant the removal of the school timetable and school setting, leaving adolescents with the demand to build structure on their own and organize their own learning. This disruption strained material resources (dedicated study spaces, clear schedules) and social resources (in‐person teacher instruction and feedback, peer study cues and encouragement). To meet these new demands, adolescents constructed replacement routines: they set fixed study periods, used planning apps, and agreed on digital check‐ins with parents or friends. For instance, Benny (11) shares his adaptive strategy:

At the beginning of school, it was like, “How am I going to get all these tasks done,” that was a big problem at first; but then my mother and I found appropriate solutions with apps where we could track the time, so that I worked every day for three hours and 30 minutes, and then I also used a checklist, which worked very well.

Others, such as Thomas (14), kept motivation high by pairing homework blocks with short online‐gaming sessions shared with classmates, while Maya (11) preferred using the phone and just having a classmate on the line while they did homework together. Parents often assisted with structuring workloads and creating a conducive learning environment at home, while older siblings were often a resource when explaining school material. These tactics drew on available material resources (internet access, devices, learning spaces at home), social support (parental instruction, motivation by parents/peers, older siblings), and personal resources (motivation), which were easier to tap into with older age. This pattern illustrates a problem‐focused coping strategy: adolescents actively modified their environment to restore normal functioning.

#### Purposeful activity and engagement

The closure of schools, sports clubs, and youth centres left many adolescents facing a new demand to fill surplus hours in meaningful ways. In the context of what we identified as an “empty‐schedule” stressor, personal resources (purpose, intrinsic motivation) were diminished, and social cues that normally structure after‐school time were absent. To counter boredom and loss of direction, many adolescents set self‐directed goals: Nikos (15) took up home fitness, Elif (12) cycled through new craft projects and books, and Jan (14) threw himself into a full bedroom renovation: “It was the biggest project I've ever done. I liked having a project or something to do”, he said, delighted by the sense of progress. Some students, like Hadiya (14), reframed coursework as a long‐term project, turning academic tasks into daily milestones. These efforts drew on material resources at home (tools, craft supplies, books, online tutorials) and personal resources (curiosity, self‐discipline), gradually restoring a sense of efficacy and forward momentum. The theme therefore again represents a problem‐focused coping strategy: adolescents generated new tasks to rebuild structure and keep active in an otherwise aimless context.

#### Social and family connections

Lockdown and physical distancing rules severed everyday face‐to‐face contact with friends, classmates, and extended family, creating a demand to keep friendships alive without physical presence. The sudden absence drained social resources and risked growing loneliness. Adolescents therefore worked to maintain, rather than replace, their friendships through digital means. Thomas (14) described leaving the game‐chat channel open after online matches so his friends could talk together. Others, such as Nikos (15) set up group calls at a fixed recurring schedule. These touch‐points preserved the familiar rhythm of peer interaction even while schools and social clubs were closed.

Strengthening family bonds also helped alleviate feelings of loneliness. Parents organized cooking sessions or film nights, while siblings served as instant companions and in some cases even as a replacement. Benny (11) noted:

Maybe my brothers then sort of replaced them [my friends], which meant that I maybe just didn't feel so alone. I don't know if it's the same with other children, but I didn't feel alone or anything like that, I was relatively well off.

These strategies mobilized material resources (smartphones, headsets, stable Wi‐Fi) and re‐activated social resources at two levels: virtual peer groups and intensified within‐family interaction. This theme sits at the intersection of both coping types. On the one hand, these adolescents are proactively finding new solutions to stay in touch during restrictions, which is indicative of problem‐focused coping. On the other hand, the emotional support gained from these interactions help manage feelings of loneliness, reflecting emotion‐focused coping.

#### Emotion regulation

Constant infection updates, isolation from peers, and the feeling of not knowing when restrictions would end was associated with a demand to manage heightened anxiety and worry. This overload corresponded with an erosion of personal resources for self‐soothing and thinned everyday social reassurance that usually comes from casual contact. To ease the tension, adolescents turned to activities that offered psychological relief without productivity pressure. Andrea (11) found calm by practising viola pieces online. Lars (11) took his mountain bike out for long rides, merging physical exertion with mental release. Others followed yoga videos, practiced breathing exercises, or stood alone on the balcony for short breathing breaks. Hadiya (14) chose brief retreats to a quiet room: “I try not to take it [bad mood and stress] out on other people. So I sometimes go into a room where I can calm down and stuff. Or go out for a bit and stuff.”

Emotional check‐ins or communication with peers and family members also helped. Nikos (15) reported that chatting with friends “distracts me from what I don't want to think about.” These tactics mobilized material resources (instruments, sports gear, private spaces), personal resources (body awareness, self‐control skills) and, when used, social resources (a friend on the line). Together they illustrate an emotion‐focused coping strategy – soothing anxiety generated by pandemic uncertainty rather than trying to change the external situation.

#### Avoidance and escapism

The same overload of uncertainty, alarming news feeds, and restricted movement sometimes overwhelmed adolescents, creating a demand to cope with rising anxiety, that also underlies emotion‐soothing and drained the same personal and social resources. Here however, some adolescents chose to tune out the pandemic altogether. With few private outlets available, many retreated into digital spaces, as Enesa (13) explained: “You spend all your time on your cell phone watching TV or simply want to sleep and seek peace and quiet. You distance yourself from people and don't want to be in crowds as often. That's how I feel.” Prolonged scrolling, binge‐watching, and late‐night gaming offered instant distraction but often disrupted sleep and daily routines. Some teens physically withdrew as well, closing bedroom doors or skipping family meals to carve out solitude. Others escaped by mentally replaying life “before Corona.” Elif (12) longed for normal classes “the way it used to be without masks.” However, such strategies were not mentioned by all interviewees, about 30% reported such behaviors. These forms of escape relied on minimal material resources – a smartphone, streaming service, headphones – and minimal social resources, reducing contact rather than seeking it. They illustrate an emotion‐focused avoidance strategy: turning away from distressing thoughts rather than tackling the situation.

#### Acceptance and perspective shift

The same prolonged uncertainty that fuelled anxiety for some adolescents prompted others to reframe the situation and look for benefits. The relentless news cycle and open‐ended restrictions still taxed personal and social resources, but these teens responded by adjusting their outlook rather than escaping. Elif (12) saw the difficulties as “surmountable challenges,” adding: “It's difficult, but you can get through it. It's different, but new things shouldn't only be seen negatively.”

Similarly, Enesa (13) found that starting each day with a positive mindset led to more positive experiences, a technique she learned from social media. Several interviewees identified unexpected gains – extra family time, a new found independence and self‐competence due to organizing schoolwork by themselves. Heike (11) valued the additional time spent with her father, emphasizing the positive aspects of family bonding due to the pandemic restrictions. This pattern of acceptance and perspective shift, while not universal, was clearly articulated by a quarter of the adolescents. This coping style drew chiefly on personal cognitive resources – optimism, flexible thinking, gratitude exercises, emerging self‐efficacy, and exemplifies meaning‐focused coping, where the situation (or specific parts of it) is reframed and reinterpreted in a more positive way or in way that the adolescents believe that they will manage to deal with the stressor.

### Ukrainian refugee findings and themes

As with the COVID‐19 group, we first provide a visual summary of the core findings for the Ukrainian refugee adolescents in Figure 3. This figure illustrates the distinct associations between the severe stressors of displacement, the different set of resources they mobilized, and the resulting focus of their coping efforts. The detailed themes that underpin this summary are explored next.

#### Adaptation and new routines

Sudden displacement entailed being uprooted from familiar schools, neighborhoods, and daily schedules. This situation created the demand to rebuild structure in an unfamiliar environment. The upheaval stripped material resources (predictable timetables, known transport routes) and social resources (teammates, dance partners) that normally anchor routine. To regain stability, many re‐established familiar activities inside Germany. For example, Nazar (15) found a semblance of his prewar life by engaging with a local football club in Germany. Similarly, Svetlana (12) continued her passion for dance by joining a dance group offered free of charge for Ukrainian refugees. Establishing new routines also involved adapting to the German way of life, such as learning local customs and navigating public transportation, which contributed to their overall adaptation and integration into German society. Many German schools implemented special Ukrainian classes, which allowed adolescents to come into contact with a peer group that felt culturally familiar. Tatjana (17) noted:

And when I went to school, I was able to communicate with these two teachers and with the children in Russian and Ukrainian. And it gave me a certain feeling of belonging and an atmosphere as if I was back in Ukraine.

These initiatives drew on several resources. It was often the host families the Ukrainian refugees were staying with or school staff that found free sports offering or social clubs, thereby relying on social resources, the initiative, and help from those more familiar with the offerings. Schools provided the material scaffolding to recreate structure and familiarity with the special Ukrainian “welcome” classes. Because adolescents acted directly on their new setting to recreate daily structure, the theme represents a problem‐focused coping strategy for adjusting to displacement while preserving cultural identity.

#### Educational continuity and future orientation

War‐related flight led to the disruption of Ukrainian adolescents' schooling, leaving unfinished courses in Ukraine and an unfamiliar system in Germany. This created a demand to restore academic progress and tackle uncertainty about the future. The break in studies eroded material resources (a stable classroom, familiar curriculum) and shook personal resources such as academic self‐efficacy. Many responded by throwing themselves into “double schooling”: attending German schools by day and logging into their online Ukrainian schooling in the afternoon. This “double schooling” itself could be another possible stressor that challenged personal resources such as time and mental energy, however the majority still chose to continue on both school paths. Aleksandra (17) explained the purpose of education for herself: “But I know that my whole family stayed behind in Ukraine, so it was very difficult. Now, it's much better that I have thrown myself into my studies.” Yuri (14) echoed the benefit: after a summer of “nothing to do” he said: “I like that I have something to do now. At school, I communicate, I have friends, I distract myself.” Education also became a way to safeguard future goals within their new German context: Christina (17) still aims to be a doctor and is already learning how to pursue that path in Germany. This coping strategy drew on material resources offered by German and Ukrainian schools (special Ukrainian classes in Germany, the option to complete Ukrainian education online), social resources (new classmates in Germany, old classmates in Ukraine, supportive teachers and online teacher instructions), and renewed personal resources—determination, motivation, future orientation. Functionally, this response is problem‐focused because adolescents act directly on the educational disruption, as well as emotion‐focused because concentrated study dampens rumination, and meaning‐focused because it gives a new sense of direction and creates a purposeful vision of the future.

#### Social connections and community

Displacement abruptly severed the Ukrainian adolescents from friends, classmates, neighbors, and (extended) family in Ukraine who had once anchored their social lives. This disrupted social resources and left the adolescents feeling isolated and lonely in the host country. To combat these feelings and foster integration, they tried to build a new circle of friends while still maintaining their relationships with friends and family left in Ukraine. However, existing contacts in Ukraine were now limited to often brief digital contact, often due to power outages. Different timetables and life circumstances between those still in Ukraine and those resettling to Germany made sustained closeness hard to maintain.

The quickest route to forging new connections was through the special Ukrainian “welcome” classes: shared language and parallel experiences facilitated friendships. The support network among the refugees aided in assimilating into the local educational system, with peers often helping each other with homework and encouraging each other in learning the German language. For example, Larissa (13) demonstrated this support by assisting her classmates with language translation in class: “When we went to the regular German class, it was very difficult because we didn't understand anything, and since I speak English, I helped the other Ukrainian classmates, I tried to translate the teacher's words.”

A second avenue was engagement with German peers, host families, and volunteers. At school the Ukrainians would visit some classes with their German peers, however this contact only worked well if the school director, staff, and teachers took the initiative and actively tried to integrate them socially, either by introducing them or organizing social events together. Multiple interviewees came into contact with German peers in sports clubs, however also here the main way that the Ukrainian refugees found the social clubs was through the initiative of host families or school staff. This reinforces the importance of social support from the host country community when it comes to social integration. Apart from that, this strategy relied heavily on the personal resources of the adolescents themselves, such as initiative, openness, confidence, and social skills in general. Because adolescents acted directly to rebuild an interaction network essential for daily life, the theme represents a problem‐focused coping strategy with the emotional payoff of reducing loneliness and fostering a sense of belonging.

#### Emotion regulation

Trauma from war, worry for loved ones still in Ukraine, the strain of adapting to a new country, and the uncertainty of their future in general were associated with a demand to quiet fear, worry, and homesickness. These intersecting stressors drained every tier of the adolescent resource pool. Personal resources (such as baseline calm, confidence, concentration) were challenged and further disrupted by trauma responses. Social resources shrank to the few relatives who had fled with them and the new friends they made were not as close to them yet as the friends they left behind. Material resources (such as a private, familiar bedroom, many personal items) had to be left behind and displacement left the families at a point needing time to build these resources up again. Against this backdrop, adolescents turned to low‐cost self‐soothing activities they could control. For instance, Anastasiya (13) turned to a variety of activities, from dancing to artistic endeavors, as a form of stress relief. She explains her passion for painting: “I simply love painting very much. It's a hobby for me. I paint, I buy canvases, paints. I paint just for myself, just any pictures. Yes, maybe to relieve some stress.” Additionally, she mentioned other distractions: “Well, first of all, friends. Second, I love sweets. I distract myself with sports, dancing, and beading.” Similarly, music was instrumental in emotional management for adolescents like Illya (17) who found solace in playing the piano, one that he could access for free at the public library. These pursuits offered the adolescents more than just a pastime; they provided a means to divert their focus from present difficulties and served as a medium for expressing and processing their emotions.

A second line of defense was emotional anchoring through close family. Veronika (13) highlighted the importance of sharing her worries about the war, instead of keeping her emotions bottled up. However, the physical distance from Ukrainian friends and family and the difficulty in getting in touch with those remaining in Ukraine were significant hurdles. Nevertheless, getting in touch was worth it, as digital communication reassured them that their loved ones were still safe. These coping strategies relied on material resources (hobby and creative supplies, musical instruments, spaces to engage in the activities), as well as needing quite a complex mobilization and understanding of personal resources (creative skills, body and emotional awareness, motivation) and also the only dependable social resource left, their family. They therefore exemplify a firmly emotion‐focused coping strategy – one that is difficult to mobilize under displacement, yet essential for stabilizing mood when external problems remain unsolved.

#### Avoidance and rumination

Ongoing war reports, fear for relatives, and the ache of homesickness produced the same internal demand to quiet distress that underlies other emotion‐focused responses – but some adolescents tried to do so by turning away rather than by active self‐soothing. With personal calm already eroded and few close peers in Germany, they reached for coping options that required little extra resource mobilization. One route was information avoidance. Nazar (15) chose to limit their exposure to news or avoid the topic of the war altogether – a method particularly helpful for managing stressful situations beyond their control. Others chose to withdraw from and avoid social interactions. Nina (14) preferred to isolate herself when experiencing negative feelings or worries: “No, I prefer to be alone, it's calmer for me. I only want to be with friends when I'm in a good mood. I don't want to show them my bad mood, so I'd rather keep it to myself.” While Nina still engaged in social interactions in general, others like Danylo (14) socially shut off. His circumstances completely eroded all his resources, so that he found no other coping options than to distance himself from old friends in Ukraine, his family and found no new connections in Germany.

This often went hand in hand with nostalgic rumination, as the same phone used to block news became a portal to the past when spending time alone. Nazar (15) shared his experience:

But when I'm in the photo gallery [of my phone] looking at old videos with my friends […] we talk about it, reminisce, laugh. […] But at home, whether I'm with new friends or alone, or when I want to go to sleep, I miss my old friends.

Yuri (14) similarly gets such intrusive thoughts as he often reflects on memories of his hometown in Ukraine, comparing it to his new life in Germany. A bit more than a quarter of the Ukrainian adolescents spoke of such behaviors. These responses relied on minimal material resources – a smartphone, headphones – and almost no social resources apart from occasional shared reminiscing with friends. They illustrate an emotion‐focused avoidance/rumination strategy: brief relief through shutting out new distress and mentally visiting what was lost. While low‐effort and immediately soothing, such responses can create isolation, however might be the only option for some whose resources feel completely depleted.

#### Positive perspective and hope

The very stressors that fuel avoidance – war trauma, worry for relatives, and an uncertain future – prompted some adolescents to reinterpret their situation and search for hope. With familiar material and social foundations gone, these adolescents mobilized the one resource still under their control: personal meaning‐making. Nazar (15) chose to focus on the good aspects of his life in Germany, reflecting on how his perspective on problems has evolved since the war began. He expresses gratitude for what he has now, rather than dwelling on past losses. Maria (16) found comforting advice from her counselor, helping her to view her situation in Germany as an opportunity amidst adversity. Veronika (13) particularly highlighted the value of maintaining a positive outlook:

The war has taught me a lot… I cherish moments with my loved ones more […] You should always have a positive attitude towards the situation, no matter what happens. Everything will be all right, you can take comfort in that thought.

By highlighting small gains and a new appreciation for the safety one has at this moment, adolescents gave chaotic circumstances a coherent, hopeful storyline. About one third of the interviewees described this strategy at the time of the interview, however it remains to be seen whether these views are still preserved after several years of ongoing war. This internal re‐appraisal drew chiefly on personal cognitive resources – optimism, future orientation, faith – and was reinforced by social resources when peers or adults echoed a forward‐looking message (conversations with friends about meaning and values, counselors highlighting progress in Germany). Because the adolescents changed the meaning of displacement rather than its material facts, the theme represents a meaning‐focused coping strategy: finding purpose and potential in adversity to restore motivation and emotional balance.

## DISCUSSION

Guided by our integrated theoretical model, our analysis illuminates the connections among stressors, resources, and coping within each crisis. First, regarding the coping strategies adolescents report when facing these crises, we identified distinct sets of themes for each group. In the German adolescent sample, we identified six main themes during the pandemic. Three were mainly problem‐focused coping responses to lost structure or peer access (adaptation & new routines; purposeful activity & engagement; social & family connections), and three were emotion−/meaning‐focused responses to chronic uncertainty and negative emotions (emotion regulation; avoidance & escapism; acceptance & perspective shift). Among the interviewed Ukrainian refugees, we also found six main themes. Three were mainly problem‐focused coping responses to an even greater loss of structure and peer connections and community altogether (adaptation & new routines; educational continuity & future orientation; social connections & community). The other three were also emotion‐/meaning‐focused responses to chronic uncertainty, negative emotions, and potential trauma (emotion regulation; avoidance & rumination; positive perspective & hope). These coping strategies were readily classifiable according to the foundational coping functions described by Lazarus and Folkman (1987). In line with a large body of previous research, our findings did not reveal entirely novel forms of coping, but rather confirmed the consistent “toolkit” adolescents draw upon. For instance, the strategies of adapting routines and using digital tools for social connection in the pandemic group mirror the findings of numerous quantitative and qualitative studies conducted during that time (e.g., Branquinho et al., 2020; Orgilés et al., 2021). Similarly, the emphasis on educational focus and family support among the refugee group is consistent with research on other displaced youth populations (e.g., Akgül et al., 2021; Betancourt et al., 2015).

Second, regarding the links among stressors, resources, and coping, our analysis revealed contrasting profiles for each group. Pandemic stressors (remote learning, idle time, domestic confinement) were associated with an erosion of material and social scaffolds while leaving legal safety intact. Adolescents therefore mobilized remaining resources – internet connection, parents, digital peer groups – to rebuild lost structure (problem focus) or to soothe uncertainty (emotion focus). This aligns with research showing that while pandemic restrictions disrupted key developmental systems (Bridgland et al., 2021; Ellis et al., 2020), adolescents with stronger protective resources, such as high self‐efficacy and robust social support, were often able to leverage these assets to avoid maladaptive outcomes (Ravens‐Sieberer et al., 2023; Shek & Chai, 2020).

In contrast, displacement stripped the refugees of all three resource tiers (personal, social, material), which left their coping responses gated by resources adolescents could still mobilize: low‐cost hobbies and close family support for emotion regulation; externally provided “welcome” classes and free club memberships for routine rebuilding; and positive reframing and hope. This experience of cumulative adversity mirrors findings from other conflict zones, where the loss of basic safety and material stability can narrow the coping repertoire, making emotion‐focused or disengagement strategies a necessary adaptation when external control is low (Cherewick et al., 2015; Masten & Narayan, 2012).

What changed between lockdown and displacement was not adolescents' basic coping repertoire but how strongly the crisis eroded the resources. Our findings thus illustrate the dynamic described by researchers like Schwarzer (2024): that resource availability sets the boundaries for coping. Similar to past research, our results show that abundance enables proactive strategies, while depletion limits adolescents to whatever options remain viable (Benight & Bandura, 2004). This finding contributes to our understanding of adolescent coping by demonstrating that the specific pattern of resource loss associated with a crisis is more illuminating than the general nature of the event itself. For example, knowing that the pandemic primarily strained social resources helps explain the focus on digital maintenance, whereas knowing that displacement caused a near‐total loss of all resource types clarifies why coping was focused on fundamental rebuilding. Thus, our analysis suggests that to support adolescents effectively, we must look beyond the type of crisis and focus on the unique resource landscape it creates.

Third, regarding the comparison of strategies across contexts, we found that while core coping functions were shared, the specific form of coping was highly context‐dependent. Four families of coping – routine rebuilding, creative/physical self‐soothing, digital peer contact, and positive re‐appraisal – appeared in both groups. The recurrence of these core strategies across different crises aligns with prior work showing that young people often draw on a set of commonly observed coping responses, including seeking connection and support from others, alongside meaning‐related strategies such as faith‐based coping, when navigating stress (Cherewick et al., 2015; Orgilés et al., 2021; Pfefferbaum et al., 2014).

Conversely, crisis‐specific strategies clearly highlighted how the unique qualities of each crisis were associated with distinct adaptive responses. For example, the pandemic's unique “resource gap” of unstructured idle time corresponded with adolescents' engagement in purposeful activities and projects as a key problem‐focused strategy to fill this void and maintain a sense of efficacy. In stark contrast, war displacement was associated with a critical need to secure future prospects. This context may help understand the intense focus on educational continuity and future orientation, including “double schooling”, among Ukrainian refugee youth. Similarly, while avoidance behaviors were present in both groups, their manifestation differed in response to the distinct threats: pandemic‐affected youth often engaged in digital escapism from information overload and the monotony of confinement within a relatively safe but restricted environment, whereas refugee youth's avoidance and rumination sometimes involved distancing from acute war‐related trauma reminders or focusing on a lost past, reflecting responses to direct, ongoing threats and profound, irretrievable losses. This divergence corroborates prior research indicating that while coping functions may be universal, the specific form they take is highly context‐dependent (Skinner & Zimmer‐Gembeck, 2007). Consistent with observations of youth in other conflict zones who prioritized culturally or contextually relevant strategies (Pfefferbaum et al., 2015), the Ukrainian adolescents in our study adapted their problem‐solving efforts to the unique “acculturative demands” of their displacement (Suárez‐Orozco et al., 2018), while pandemic‐affected youth adapted theirs to the digital resources available at home (Branquinho et al., 2020). Thus, similarity or divergence in coping appeared to relate not just to which resources were eroded, but on how the specific qualities of each crisis were associated with different resource landscapes and adaptive demands.

### Developmental tasks during crisis

Our side‐by‐side analysis can be further contextualized by considering core adolescent developmental tasks, such as forging mutually supportive peer relationships, negotiating behavioral and emotional autonomy within the family, and building a coherent sense of identity. The two crises appeared to disrupt these developmental tasks in different ways. In this light, the coping strategies we identified can be understood as adolescents' purposeful efforts to bridge the gaps created by each crisis. By employing these strategies, adolescents sought to protect, maintain, or resume their progress on these essential developmental goals. The specific nature of the disruption to a developmental task was thus linked to the specific constellation of coping that followed.

During lockdown, friendship circles remained intact but were pushed onto screens; the immediate risk was emotional thinning, loneliness, and fear‐of‐missing‐out. The interviewed adolescents during the pandemic responded with digital maintenance – fixed group calls, shared gaming sessions, study‐while‐chat routines – problem‐focused efforts that preserved reciprocity until in‐person contact returned. For the Ukrainian refugees, displacement severed whole networks overnight. Language barriers slowed entry into German peer groups, so initial ties were formed mainly with other newcomers in “welcome” classes. Here coping moved beyond preservation to construction: deliberate network‐building through shared classes and host‐family‐sponsored sports clubs. In both contexts, these coping efforts directly served the developmental task of building and maintaining supportive peer relationships, despite the differing obstacles each crisis presented.

Lockdown shrank decision space: parents were always present, and public life was closed. Adolescents carved out micro‐autonomy by designing their own study timetables and projects – small, problem‐focused acts that re‐established control. Displacement produced a different mix. Fathers were often still in Ukraine, paperwork and housing were managed by adults, and, crucially, close peers were not yet available. Many teens therefore leaned more heavily on mothers or older siblings for daily emotion regulation – temporarily reversing the usual drift toward emotional independence. At the same time, they gained behavioral autonomy as their German skills soon surpassed those of their parents thereby translating for parents, dealing with bureaucracy, and organizing dual schooling by themselves. Thus, dependence and independence advanced side‐by‐side. The coping strategies reported here can therefore be seen as adaptive tools used by adolescents to navigate the unique and often contradictory challenges that each crisis posed to the developmental task of achieving autonomy.

Adolescents during the pandemic faced postponed milestones that normally scaffold future identity. Their answer relied on meaning‐focused strategies such as acceptance and perspective‐shift: some reframed the hiatus as a chance to discover new interests, others practiced gratitude for health or family time. For the Ukrainian group, the challenge was not delay but dual belonging. Adolescents suddenly inhabited two cultural worlds, spoke two languages, were enrolled in two school systems and, in many cases, lived apart from fathers or extended family, creating a tension central to acculturation theory (Berry et al., 2006). They continued to feel connected with their homeland, but they also started to see Germany as a second home and started planning their futures in Germany. This hybrid‐identity created tension, the need for preserving Ukrainian culture while integrating into German society. This effort to value both cultures aligns with what acculturation theory describes as an “integration” strategy (Berry et al., 2006). Positive re‐appraisal and cultural peer bonding reframed the tension as an opportunity rather than a loss. Here, coping served as key tools for addressing the distinct threats to identity formation that each crisis presented.

### Practical implications

Our findings show that adolescent coping is only as strong as the resources remaining within reach. Effective support must therefore protect or restore material, social, and personal assets before problem‐, emotion‐ or meaning‐focused strategies can be expected to succeed.

On the material side, private space and reliable internet proved critical: both German and refugee youths could rebuild routines or maintain peer ties only when they had a quiet corner and stable connectivity. For refugees, secure legal status and housing were the fundamental first steps. Ukrainian students benefited from EU‐wide protection that granted immediate residency and access to regular schools – support not available to many other refugee groups. “Welcome classes” further met urgent needs by accelerating language learning and offering contact with peers who shared their cultural background, while free sports‐club memberships and free access to cultural activities helped displaced adolescents graft new routines onto German life.

School contingency plans should be able to facilitate devices to their students, but also consider providing study/meeting rooms for families without spare rooms, given the importance in the interviews. Replicating that fast‐track legal certainty and predictable housing is a precondition for supporting any future refugee adolescents. Cost‐free extracurricular activities or similar low‐barrier schemes would help other refugee and low‐income local youths alike; information about such programmes must also be shared proactively, as a lack of awareness can block uptake.

Social resources mattered just as much. During lockdown, adolescents kept friendships alive through digital maintenance, yet emotional depth thinned. Among Ukrainian refugees, welcome classes created an initial peer group, but genuine integration required schools to facilitate regular contact with German classmates. Schools thus need to treat social (re)connection as part of their educational mandate. Structured peer‐reconnection should be prioritized after lockdowns or long periods in which adolescents cannot meet in person. For refugee integration this mandate is even greater, as schools must create an inclusive, accepting atmosphere, supported by deliberate social‐learning activities. Furthermore, affirming cultural heritage is essential to mitigate bullying and promote belonging.

Adolescents who already possessed a strong repertoire of personal resources – self‐efficacy, optimism, motivation, confidence – handled both crises more easily and could deploy a wider coping toolkit. By contrast, several Ukrainian interviewees showed trauma reactions or possible PTSD symptoms that can drain or block personal resources if left untreated. Strengthening personal resources in general is valuable, yet acute trauma must be a priority for healthcare professionals, with culturally sensitive assessment and timely intervention.

### Strengths, limitations, and future research

This study's primary strength lies in its comparative approach, by analyzing two qualitatively different crises within a single analytic frame, we could explore the connections among specific stressors, resource depletion, and reported coping strategies. To our knowledge, no previous work has qualitatively illuminated this stressor‐resource‐coping dynamic across both a pandemic lockdown and war‐related displacement. The use of in‐depth, semi‐structured interviews added further value, allowing adolescents to express their experiences and coping mechanisms in their own words.

However, limitations include reliance on secondary analysis of existing data not initially aimed at exploring every aspect of the stressor‐resource‐coping dynamic, so some strategies may have gone unreported. Furthermore, our single‐interview format captures a snapshot in time. It does not allow us to explore the dynamic process of re‐appraisal, where an adolescent's perception of a stressor might change over time as a result of successful coping, a point that a longitudinal design could illuminate. Additionally, while the first author's native fluency in Ukrainian and German ensured a careful and conceptually equivalent translation of the Ukrainian interviews, a formal back‐translation process was not conducted. This is a limitation, as this process could have further verified that subtle cultural nuances were not lost in translation. Considering the Ukrainian sample was interviewed six to eight months after arrival in Germany, reactions during the acute flight phase and the resource cascade that follows remain undocumented. Additionally, comparing coping strategies between groups is complex, especially since Ukrainian refugees had prior exposure to the pandemic, which might have influenced their appraisal and coping responses compared to those only experiencing the pandemic. Cultural biases also pose a challenge, as coping experiences can vary widely across different cultural backgrounds. Furthermore, both samples lived in a high‐income country with an intact digital infrastructure; the findings may not generalize to settings where crises threaten physical safety and eliminate internet access (e.g., climate‐related disasters or conflicts with ongoing shelling). Finally, cultural and policy contexts limit transferability: Germany's swift legal protection for Ukrainian refugees and openness to Ukrainian refugees provided support that many other refugee populations do not receive.

Future research should delve into the long‐term effects of different coping strategies on resilience and psychological well‐being. Prospective, multiwave studies that track stressors, resource shifts, and coping choices—together with baseline and follow‐up mental‐health indices—would clarify which early responses bolster, and which undermine, later adjustment. Such evidence will sharpen intervention targets and help practitioners foster coping patterns that sustain adolescents' well‐being both in the immediate crisis and over the years that follow.

## CONCLUSION

This study aimed to explore and compare adolescent coping strategies during the COVID‐19 pandemic lockdown and war‐related displacement, using an integrated theoretical framework to analyze the connections among contextual stressors, resource availability, and coping responses. Our reflexive thematic analysis identified both shared coping functions – such as regulating emotions and maintaining social connections – and distinct, crisis‐specific strategies.

Crucially, the specific coping strategies adolescents reported were closely associated with the unique resource landscapes of each crisis. Pandemic‐affected youth largely mobilized existing resources to adapt to strained routines. Displaced Ukrainian youth, on the other hand, faced a profound loss of personal, social, and material resources, which was linked to coping efforts focused on fundamental rebuilding and navigating acculturative demands. These findings underscore that adolescent coping is a dynamic process contingent on the availability of protective resources.

Theoretically, these findings demonstrate the value of integrating transactional and risk‐resilience models to explore how the stressor‐resource‐coping dynamic operates with both underlying commonalities and distinct variations across qualitatively different crises. Practically, the results suggest that recognizing the specific resource gaps created by a crisis is essential for developing tailored interventions. Ultimately, supporting adolescent well‐being in crisis requires a focus on protecting and restoring the critical resources that underpin effective coping.

## AUTHOR CONTRIBUTIONS

**Sophia Chabursky:** Conceptualization; methodology; writing – original draft; writing – review and editing; formal analysis. **Sabine Walper:** Supervision; validation; conceptualization.

## FUNDING INFORMATION

This research received no specific grant from any funding agency in the public, commercial, or not‐for‐profit sectors.

## CONFLICT OF INTEREST STATEMENT

The authors declare that there are no conflicts of interest regarding the publication of this paper.

## ETHICS STATEMENT

This study is a secondary analysis of data from two prior qualitative studies conducted at the German Youth Institute. Although formal ethics approval was not obtained for the initial studies, as the ethical guidelines of the institute had not yet been formalized into an ethics committee at that time, all conducted studies were required to adhere strictly to the institute's ethical standards. These standards align with the 1964 Helsinki Declaration and its later amendments, or comparable ethical standards. Detailed information on the ethical procedures is outlined in the following paragraphs.

Ethics and methodological details: The initial data collection was conducted with full respect to the principles of informed consent, participant anonymity, and well‐being. In both studies, informed consent was explicitly secured for future research use from all participants. For minors, informed consent was additionally obtained from parents or legal guardians, ensuring compliance with ethical guidelines. Participants were informed at the beginning of the interviews that they could opt out of answering any question or terminate the interview at any point without any adverse consequences.

Special attention was paid to the emotional well‐being of the participants, particularly during interactions with vulnerable groups such as Ukrainian refugees. Interviewers were trained to sensitively manage interviews and discussions, refraining from probing into sensitive subjects when deemed potentially distressing (such as trauma and war experiences). Participants were also provided with information about available psychological support services, should they experience distress following the interviews.

Informed consent forms: The informed consent forms utilized in the original studies detailed the measures taken to protect personal data according to the European Union's General Data Protection Regulation (GDPR) and the Federal Data Protection Act (BDSG). These forms, which were comprehensively explained to participants and their legal guardians, covered the purpose and scope of the data processing, the voluntary nature of participation, and the right to withdraw consent at any time without disadvantage. These documents stated that the collected personal data would be used exclusively for scientific research purposes and not for commercial use. Recordings from interviews were anonymized as soon as practicable to prevent any identification of participants from the recorded data, in accordance with the commitment to participant confidentiality and data security detailed in the consent forms.

Both studies were overseen by the German Youth Institute, which was responsible for ensuring compliance with data protection regulations and ethical standards. Data were handled by trained personnel committed to confidentiality and were not disclosed to unauthorized third parties.

This comprehensive approach to ethical compliance in the initial data collection ensures the integrity of the data used in this secondary analysis. The detailed methods and informed consent procedures employed substantiate the ethical foundation of our study, compensating for the absence of formal ethics committee approval at the time of data collection.

## Supporting information


**Table S1.** Detailed findings of the analysis: stressors, resource impacts, and coping strategies.

## Data Availability

The interview data generated and analyzed during this study are held in a repository managed by the German Youth Institute, where the original research was conducted. Due to ethical and confidentiality constraints, restrictions apply to the availability of these data, which were used under the institute's policy for this study. While the data are not publicly available, they can be accessed upon reasonable request and with the permission of the German Youth Institute subject to institutional approvals. Interested researchers can contact the corresponding author, who will facilitate the request for data access in accordance with the institute's policies.
