# Supplementary material for: Exploring coping strategies among adolescents during COVID‐19 and war displacement: A qualitative analysis comparing two crisis settings
Source: J Res Adolesc. 2026 Feb 15;36(1):e70150. doi: 10.1111/jora.70150 (PMC12906691; doi:10.1111/jora.70150)
Supplement: Supplementary file 1 — Table S1. Detailed findings of the analysis: stressors, resource impacts, and coping strategies. [file JORA-36-0-s001.pdf]

Table A1

Findings of the Analysis

	Themes	Associated Contextual Stressor	Associated Impact on Resources	Resources Associated with Coping	Prevalence
**COVID-19-Pandemic**	Adaptation and New Routines	Sudden shift to remote learning; loss of school timetable & setting; demand to build structure and organize own learning.	material: dedicated study spaces, clear schedules;<br>social: in-person teacher instruction/feedback, peer study cues/encouragement	material: internet access, devices, learning spaces at home;<br>social: parental/sibling instruction, motivation by parents/peers;<br>personal: motivation	20/20
	Purposeful Activity & Engagement	Closure of schools/clubs/youth centres; demand to fill surplus hours meaningfully; "empty-schedule" stressor.	social: peer contact as cues for after-school time;<br>personal: purpose, intrinsic motivation;	material: supplies at home;<br>personal: curiosity, self-discipline, efficacy, forward momentum	20/20
	Social and Family Connections	Lockdown & physical distancing severing face-to-face contact; demand to keep friendships alive without physical presence.	social: everyday face-to-face contact with friends/classmates/extended family, risk of loneliness	material: internet access, devices;<br>social: virtual peer groups, intensified within-family interaction/companionship	20/20
	Emotion Regulation	Overload of uncertainty, alarming news feeds, peer isolation and restricted movement; demand to manage heightened anxiety.	social: everyday social reassurance from peer contact;<br>personal: self-soothing capacity	material: hobby supplies, private spaces;<br>social: peer/family emotional check-ins/distraction;<br>personal: creative skills, body awareness, self-control skills	15/20

	Avoidance and Escapism	Overload of uncertainty, alarming news feeds, peer isolation and restricted movement; demand to manage heightened anxiety.	social: everyday social reassurance from peer contact;<br>personal: self-soothing capacity	material: internet access, devices, private spaces;<br>social: minimal - contact reduced rather than sought out	6/20
	Acceptance and Perspective Shift	Overload of uncertainty, alarming news feeds, peer isolation and restricted movement; demand to manage heightened anxiety.	social: everyday social reassurance from peer contact;<br>personal: self-soothing capacity, sense of control	personal: optimism, flexible thinking, gratitude, emerging self-efficacy	5/20
**Ukraine-War Displacement**	Adaptation and New Routines	Sudden displacement; unfamiliar schools, neighbourhoods, daily schedules; demand to rebuild structure in new environment.	material: homes and financial resources, predictable timetables, known transport routes;<br>social: local peer network	material: inclusion in the German school system, free access to sports/social clubs;<br>social: host families, school staff finding offers, peer groups in “welcome” classes	25/25
	Educational Continuity and Future Orientation	Shattered schooling (unfinished grade, unfamiliar system); demand to restore academic progress & certainty about future.	material: stable classroom, familiar curriculum;<br>personal: academic self-efficacy	material: inclusion in the German school system, Ukrainian online classes, internet access, devices;<br>social: new/old classmates, supportive teachers;<br>personal: determination, motivation, future orientation	25/25
	Social Connections and Community	Displacement severing ties to friends/family in Ukraine; isolation/loneliness in host country; demand to build new networks.	social: established friendships, classmates, neighbors, extended family in Ukraine	material: internet access, devices;<br>social: Ukrainian “welcome” classes, German peers/host families/volunteers, family who fled with them;	25/25

			personal: initiative, openness, confidence, social skills	
Emotion Regulation	War trauma, ongoing war reports, worry for loved ones, strain of adapting, future uncertainty; demand to quiet fear, worry, homesickness.	material: familiar private spaces, personal items left behind<br>social: shrank to few relatives/new friends not as close;<br>personal: baseline calm, confidence, concentration - challenged by trauma	material: hobby supplies, accessible/ private spaces;<br>social: close family support, digital contact with loved ones in Ukraine;<br>personal: creative skills, body awareness, self-control skills	18/25
Avoidance and Rumination	War trauma, ongoing war reports, worry for loved ones, strain of adapting, future uncertainty; demand to quiet fear, worry, homesickness.	material: familiar private spaces, personal items left behind<br>social: shrank to few relatives/new friends not as close;<br>personal: baseline calm, confidence, concentration	material: internet access, devices;<br>social: minimal - contact reduced/avoided rather than sought out	7/25
Positive Perspective and Hope	War trauma, ongoing war reports, worry for loved ones, strain of adapting, future uncertainty; demand to quiet fear, worry, homesickness.	material: familiar private spaces, personal items left behind<br>social: shrank to few relatives/new friends not as close;<br>personal: baseline calm, confidence, concentration	social: reinforcement from peers/adults echoing forward-looking messages;<br>personal: optimism, future orientation, faith, meaning-making	8/25
